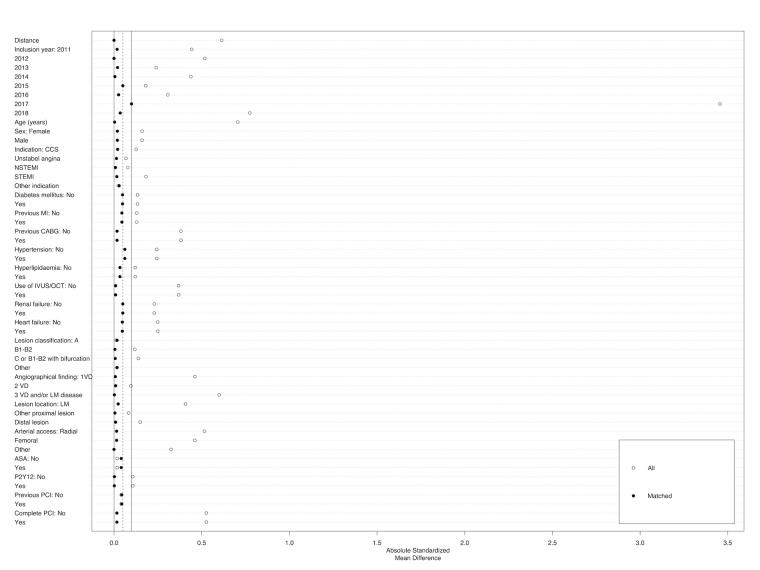# Long-Term Outcome of Percutaneous Coronary Intervention Using Absorb Bioresorbable Scaffold: A SCAAR Study

**DOI:** 10.1016/j.jscai.2025.103724

**Published:** 2025-07-29

**Authors:** Saman Saidi-Seresht, Sacharias von Koch, David Erlinge, Stefan James, Sasha Koul, Per Grimfjärd

**Affiliations:** a Department of Cardiology, Västerås Hospital, Västerås, Sweden; b Department of Medical Sciences, Cardiology, Uppsala University, Uppsala, Sweden; c Department of Cardiology, Clinical Sciences, Lund University, Skane University Hospital, Lund, Sweden; d Uppsala Clinical Research Center, Uppsala, Sweden

**Keywords:** absorb, bioresorbable scaffold, percutaneous coronary intervention

## Abstract

**Background:**

Bioresorbable scaffolds have been associated with inferior outcomes compared to contemporary permanent metallic drug-eluting stents (DES) for percutaneous coronary intervention, particularly within the initial years after implantation; however, their long-term performance remains uncertain. This study aimed to evaluate the long-term outcomes of Swedish patients treated with Absorb bioresorbable scaffolds (Abbott) vs contemporary DES, assessing device-related complications and examining potential late benefits. The findings seek to clarify the balance between early risks and long-term advantages of bioresorbable scaffolds in clinical practice.

**Methods:**

Complete data from the Swedish Coronary Angiography and Angioplasty Registry (SCAAR) was used to identify all patients receiving Absorb bioresorbable scaffolds or contemporary DES from November 4, 2011 to March 2, 2018. After 1:2 propensity score matching against modern DES, stent thrombosis, target lesion revascularization, in-stent restenosis, myocardial infarction, and all-cause mortality were analyzed. Landmark analyses were performed from 3 years onward. All patients were followed until January 17, 2022.

**Results:**

Among 1960/2406 propensity score matched patients/stents (583/802 Absorb bioresorbable scaffolds and 1377/1604 contemporary DES), bioresorbable scaffolds were associated with significantly higher early stent thrombosis, target lesion revascularization, and in-stent restenosis rates. All-cause mortality and myocardial infarction rates did not differ significantly over the entire follow-up. Beyond 3 years, the device-related outcomes converged, while myocardial infarction rates were lower with Absorb bioresorbable scaffolds than contemporary DES.

**Conclusions:**

Absorb bioresorbable scaffolds showed inferior early clinical performance compared with contemporary DES, but after 3 years, device-related outcomes were similar, while myocardial infarction rates favored Absorb bioresorbable scaffolds. These findings suggest a complex trade-off between early device-related events and potential long-term benefits of bioresorbable scaffold-mediated vascular restoration.

## Introduction

Bioresorbable scaffolds were developed to improve the long-term outcomes of permanent metallic drug-eluting stents (DES), including in-stent restenosis (ISR) and stent thrombosis (ST). The most extensively studied and used device, Absorb everolimus-eluting bioresorbable scaffold (BRS; Abbott), initially showed promising results, but extended follow-up revealed inferior outcomes compared with contemporary DES, notably higher target lesion revascularization (TLR) and ST rates.^1^, ^2^, ^3^ Efforts to refine BRS implantation techniques with meticulous predilatation, high-pressure postdilatation, and the use of intravascular imaging improved outcomes, but the absolute rates of TLR at 5 years remained higher for BRS than DES (17.5% vs 14.5%), and ST also persisted at a higher rate (1.7% vs 1.1%).^4^^,^^5^ Interestingly, from 3 to 5 years postimplantation, when complete scaffold resorption is expected, the ABSORB IV trial reported comparable TLR and device thrombosis rates between BRS and DES.^3^ These findings imply that while early performance of BRS may be inferior, a "catch-up" occurs later as the scaffold resorbs. Still, there is limited data on outcome after BRS implantation beyond 3 years. In this study, we aimed to evaluate the long-term outcomes of BRS compared with contemporary DES in a comprehensive, real-world national population from the Swedish Coronary Angiography and Angioplasty Registry (SCAAR).

## Materials and methods

### Data source

SCAAR, part of the national Swedish Web-system for Enhancement and Development of Evidence-based care in Heart Disease Evaluated According to Recommended Therapies (SWEDEHEART), holds comprehensive data on all coronary angiographies and percutaneous coronary intervention (PCI) performed in Sweden. The collected data include patient demographic characteristics, comorbidities, lesion characteristics, procedural details, pharmacotherapy, and subsequent events.^6^ Mandatory reporting on previously implanted stents and treated segments facilitates robust surveillance of clinically driven ISR and ST. The registry provides complete follow-up on mortality, myocardial infarction (MI), and repeat PCI, ensuring reliable long-term outcome assessment.

### Patient population

**Figure 1:**
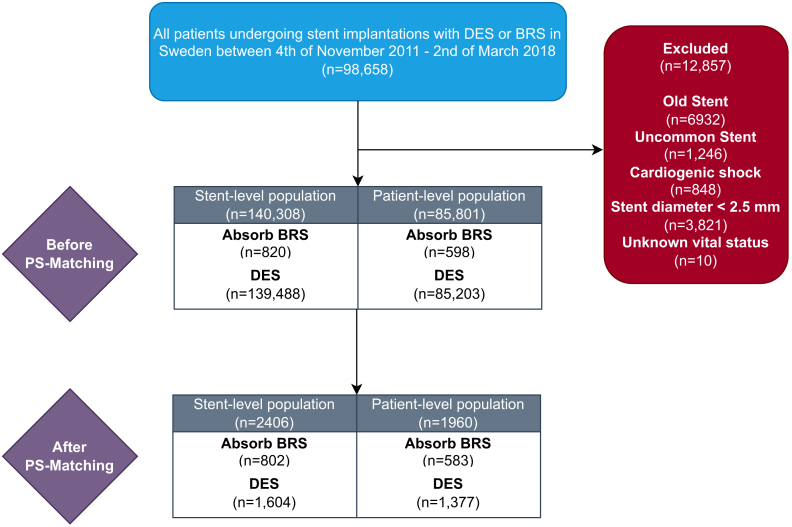
**Study flow chart.** Population before and after PS matching. BRS, Absorb everolimus-eluting bioresorbable scaffold; DES, drug-eluting stent; PS, propensity score.

All patients undergoing PCI with DES or BRS implantation in Sweden from November 4, 2011 to March 2, 2018 were identified. Patients treated with old stents, including bare metal stents (n = 6877) and noncontemporary DES (n = 55), as well as uncommon DES or DES with diameters <2.5 mm and patients with cardiogenic shock were excluded. The index procedure during the study period was considered the inclusion date. Each patient was followed up until January 17, 2022. The median follow-up time was 7.3 years (IQR, 6.4-8.0 years). The longest follow-up time was 10.2 years. The study flow chart is presented in Figure 1.

### Statistical methods

Histograms were used to assess distribution of continuous variables. Normally distributed continuous variables were assessed using independent *t* test and are presented as mean with SD. Nonnormally distributed variables were assessed with Mann-Whitney *U* test and are presented as median with IQR. Categorical variables are presented as count with percentage and difference and were assessed using the χ^2^ test. Propensity score (PS) matching using the stent-level cohort was employed to address confounding bias by balancing the distribution of baseline characteristics across the BRS and DES patient groups. PS matching simulates some aspects of randomization by pairing patients with similar propensity scores (probabilities of receiving treatment based on observed baseline covariates). Two-to-one PS matching was carried out with the nearest-neighbor approach using a no-replacement caliper method with caliper 0.2, providing precise matches and ensuring closely comparable patient groups. Patients were matched on inclusion year, age, sex, indication (chronic coronary disease, ST-elevation MI, non-ST-elevation MI, unstable angina, and other indications), diabetes mellitus, previous MI, previous PCI, previous coronary artery bypass surgery, hypertension, hyperlipidemia, renal failure, heart failure, aspirin prior to PCI, P2Y12 inhibitor prior to PCI, use of intracoronary imaging (intravascular ultrasound/optical coherence tomography), American College of Cardiology/American Heart Association lesion classification (A1, B1, B2, and C, with or without bifurcation), angiographic findings (1-vessel disease, 2-vessel disease, and 3-vessel and/or left main disease), lesion location (left main lesion, other proximal lesion, or distal lesion), arterial access (radial, femoral, and other), and complete revascularization. Covariates were carefully selected a priori to mitigate bias resulting from confounders, enhancing the internal validity and comparability between treatment groups. The effectiveness of the matching procedure was verified through standardized differences (standardized percentage bias) between matched groups, visually illustrated through balance plots (Supplemental Table S1). Covariates were considered adequately balanced if the standardized mean differences were <10%, reflecting successful confounder control. The proportion of missing values in variables of interest was low (Supplemental Table S1).

### Outcome analysis statistical methods

The comparative outcome analysis of BRS vs DES was conducted using univariable Cox regression models and Kaplan–Meier estimates for the PS-matched cohort. Hazard ratios (HR) with corresponding 95% CI and *P* values were reported from Cox regression analyses. Clinical outcomes including ST, TLR, and ISR were assessed at the stent-level, treating each stent as a unique observation. To account for within-patient clustering and reduce bias, we employed a Cox proportional hazards model, due to its ability to account for time-to-event data and censored observations common in long-term follow-up studies, with lesions nested on patient level. After the stent-level analysis, data were aggregated at the patient level to evaluate all-cause mortality, MI, and any restenosis. Any restenosis was defined as >50% narrowing within a previously implanted stent, assessed visually by angiography or by invasive methods (pressure-wire based or by imaging) during coronary angiography performed for any clinical indication. Clinical outcomes were monitored through January 17, 2022. Subgroup analyses were performed based on sex, age (≥70 years vs <70 years), diabetes mellitus status, and the use of intracoronary imaging. The subgroups were examined for ST and MI using univariable Cox proportional regression models, with interaction *P* values calculated to assess effect modification. A landmark analysis evaluated outcomes beyond 3 years. The proportional hazard assumption, that the HR between groups remains constant over time, was verified by comparing estimated log-log survival curves for parallelism for each outcome. Visual assessment of these curves was employed such that nonintersecting and parallel curves indicate compliance with this assumption. No substantial violations were observed, confirming that Cox regression was an appropriate analytical choice for this study. Additionally, the analysis accounted for clustering at the patient level, as multiple stents might be implanted within a single patient. A clustered Cox regression (with lesions nested within patients) was applied to ensure valid inference in the presence of potential correlations among repeated measurements or procedures in the same individuals. The landmark analysis was conducted to evaluate outcomes specifically beyond 3 years, conditional on patients remaining event-free up to that time point. This method addresses the temporal change in risk profiles, focusing on the long-term comparative effectiveness of BRS vs DES after the early scaffold resorption period and expected vascular restoration. Landmark analysis thus provided critical insights into potential late benefits or persistent risks that might not be visible in traditional cumulative analyses. All analyses were based on complete case data, given the low proportion of missing values for key variables. Statistical significance was defined as a 2-sided *P* value of < .05 without adjustment for multiplicity. PS matching was conducted using R version 4.2.2 (The R Foundation for Statistical Computing; package MatchIt). Data management and all remaining statistical analyses were completed using STATA/SE version 17.0 (StataCorp LLC).

## Results

### Background data

The final study population included more than 140,000 stents, comprising more than 800 BRS and nearly 140,000 commonly used contemporary DES. This represented approximately 86,000 patients, including nearly 600 with BRS and more than 85,000 with DES.

Baseline patient characteristics before PS matching are outlined in Supplemental Table S1. Patients treated with BRS were younger, with a mean age of 60 years compared to 68 years in the DES group, and predominantly men (80% vs 74% in the DES group). BRS-treated patients had previous stroke, MI, and coronary artery bypass surgery less often than DES-treated patients. The indication for PCI was less often ST-elevation MI and more frequently chronic coronary disease for patients receiving BRS than DES. Left main and 3-vessel disease was less prevalent with BRS. Moreover, mean stent diameter was greater, mean stent length shorter, and post dilation was more often performed with BRS. Arterial access was more often radial and intracoronary imaging used more frequently with BRS.

A 1:2 PS-matched study population included 2406 stents (802 BRS and 1604 DES) placed in 1960 patients, comprising 583 BRS and 1377 DES recipients. Baseline patient characteristics after PS matching were similar between the BRS and DES groups, as shown in Tables 1 and 2.

**Table 1:** Patient-level baseline characteristics after propensity score matching.

Characteristics	DES (n = 1377)	Absorb BRS (n = 583)	*P*
Inclusion year			.61
2011-2013	404 (29.3%)	161 (27.6%)	
2014	523 (38.0%)	219 (37.6%)	
2015-2018	450 (32.7%)	203 (34.8%)	
Age, y	60.1 ± 11.0	60.3 ± 11.2	.65
≥80 y	53 (3.8%)	23 (3.9%)	.92
Men	1121 (81.4%)	468 (80.3%)	.56
Smoking status			.12
Non-smoker	553 (41.2%)	245 (43.4%)	
Previous smoker	448 (33.4%)	201 (35.6%)	
Active smoker	342 (25.5%)	119 (21.1%)	
Comorbidities			
Diabetes mellitus	220 (16.0%)	100 (17.2%)	.52
Hypertension	294 (21.4%)	133 (22.8%)	.47
Hyperlipidemia	569 (41.3%)	258 (44.3%)	.23
Heart failure	31 (2.3%)	16 (2.7%)	.51
Renal failure	8 (0.6%)	6 (1.0%)	.28
Estimated GFR, mL/min/1.73 m²	88.9 ± 21.3	88.3 ± 22.7	.63
Previous stroke	47 (3.4%)	19 (3.3%)	.86
Previous MI	159 (11.5%)	80 (13.7%)	.18
Previous PCI	187 (13.6%)	89 (15.3%)	.33
Previous CABG	25 (1.8%)	11 (1.9%)	.91
Procedural characteristics			
Indication			.95
Chronic coronary disease	387 (28.1%)	158 (27.1%)	
Unstable angina	277 (20.1%)	118 (20.2%)	
Non-STEMI	389 (28.2%)	174 (29.8%)	
STEMI	267 (19.4%)	111 (19.0%)	
Other	57 (4.1%)	22 (3.8%)	
Contrast volume, mL	169.5 ± 79.0	150.4 ± 72.8	< .001
Arterial access			.35
Radial	1296 (94.1%)	539 (92.5%)	
Femoral	78 (5.7%)	43 (7.4%)	
Other	3 (0.2%)	1 (0.2%)	
Use of intracoronary imaging	259 (18.8%)	110 (18.9%)	.98
Angiographic findings			.81
1-Vessel disease	898 (65.2%)	386 (66.2%)	
2-Vessel disease	359 (26.1%)	144 (24.7%)	
3-Vessel disease and/or left main disease	120 (8.7%)	53 (9.1%)	
No. of stents	1.87 ± 1.08	1.76 ± 1.67	.04
Complete revascularization	1188 (86.3%)	512 (87.8%)	.36
Medications prior to PCI			
Aspirin	451 (32.8%)	204 (35.0%)	.34
P2Y12 inhibitor	153 (11.1%)	61 (10.5%)	.67
Statin	431 (31.3%)	203 (34.8%)	.13
β-blocker	400 (29.0%)	190 (32.6%)	.12
ARB/ACEi	377 (27.4%)	161 (27.6%)	.91
Calcium channel blocker	201 (14.6%)	74 (12.7%)	.27

Values are n (%) or mean ± SD.

ACEi, angiotensin-converting enzyme inhibitors; ARB, angiotensin receptor blocker; BRS, bioresorbable scaffold; CABG, coronary artery bypass graft surgery; DES, drug-eluting stents; GFR, glomerular filtration rate; MI, myocardial infarction; PCI, percutaneous coronary intervention; STEMI, ST-elevation myocardial infarction.

**Table 2:** Stent-level baseline characteristics after propensity score matching.

Characteristics	DES (n = 1604)	Absorb BRS (n = 802)	*P*
Lesion location			.82
Left main lesion	7 (0.4%)	5 (0.6%)	
Other proximal lesion	564 (35.2%)	283 (35.3%)	
Distal lesion	1033 (64.4%)	514 (64.1%)	
Lesion classification			.91
Type A	136 (8.5%)	73 (9.1%)	
Type B1-B2	1052 (65.6%)	524 (65.3%)	
Type C or B1-B2 with bifurcation	415 (25.9%)	204 (25.4%)	
Other	1 (0.1%)	1 (0.1%)	
Stent diameter, mm	3.1 ± 0.5	3.3 ± 0.4	< .001
Stent length, mm	20.8 ± 8.0	19.9 ± 6.1	.007
Use of postdilatation	578 (36.0%)	517 (64.5%)	< .001
Stent			< .001
BioMatrix (Biosensors)	52 (3.2%)	0	
Xience Prime (Abbott)	16 (1.0%)	0	
Promus Element (Boston Scientific)	23 (1.4%)	0	
Resolute Integrity (Medtronic)	367 (22.9%)	0	
Orsiro (Biotronik)	107 (6.7%)	0	
Absorb BRS (Abbott)	0	802 (100%)	
Promus Element Plus (Boston Scientific)	60 (3.7%)	0	
Xience Xpedition (Abbott)	164 (10.2%)	0	
Promus Premier (Boston Scientific)	400 (24.9%)	0	
Synergy (Boston Scientific)	185 (11.5%)	0	
Ultimaster (Terumo)	37 (2.3%)	0	
Biofreedom (Biosensors)	3 (0.2%)	0	
Resolute Onyx (Medtronic)	181 (11.3%)	0	
Xience ProX (Abbott)	9 (0.6%)	0	

Values are n (%) or mean ± SD.

BRS, bioresorbable scaffold; DES, drug-eluting stents.

### Outcome, full study period

**Figure 2:**
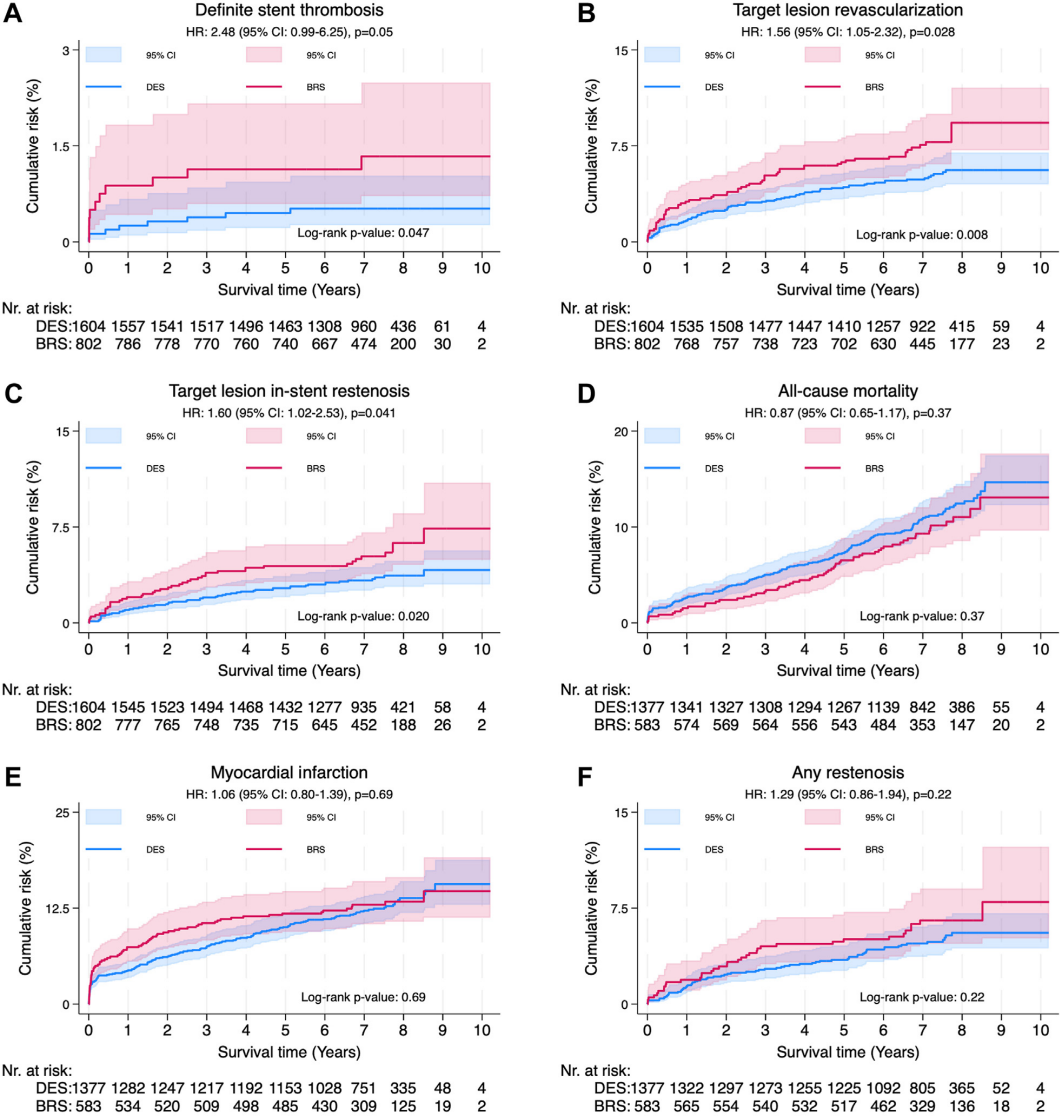
**Full study period outcomes.** Kaplan–Meier curves, outcome. Panels **A**-**F** show Kaplan-Meier event curves for ST, TLR, ISR, all-cause mortality, MI, and any restenosis respectively. BRS, Absorb everolimus-eluting bioresorbable scaffold; DES, drug-eluting stent; HR, hazard ratio.

Figure 2A-C shows Kaplan–Meier event curves for ST, TLR, and ISR. Event rates were significantly higher in patients receiving BRS than DES (ST: HR, 2.48; 95% CI, 1.03-5.98; *P* = .05, log-rank *P* = .047; TLR: HR, 1.56; 95% CI, 1.05-2.32; *P* = .028; ISR: HR, 1.60; 95% CI, 1.02-2.52; *P* = .041). The divergence occurred early and persisted throughout the study period.

Figure 2D-F displays Kaplan–Meier curves for all-cause mortality, MI, and any restenosis. No statistically significant differences were observed between patients treated with BRS and DES (all-cause mortality: HR, 0.87; 95% CI, 0.65-1.18; P = .37; MI: HR, 1.06; 95% CI, 0.81-1.39; *P* = .69; any restenosis: HR, 1.29; 95% CI, 0.86-1.94; *P* = .22).

### Outcome, landmark analysis after 3 years

**Figure 3:**
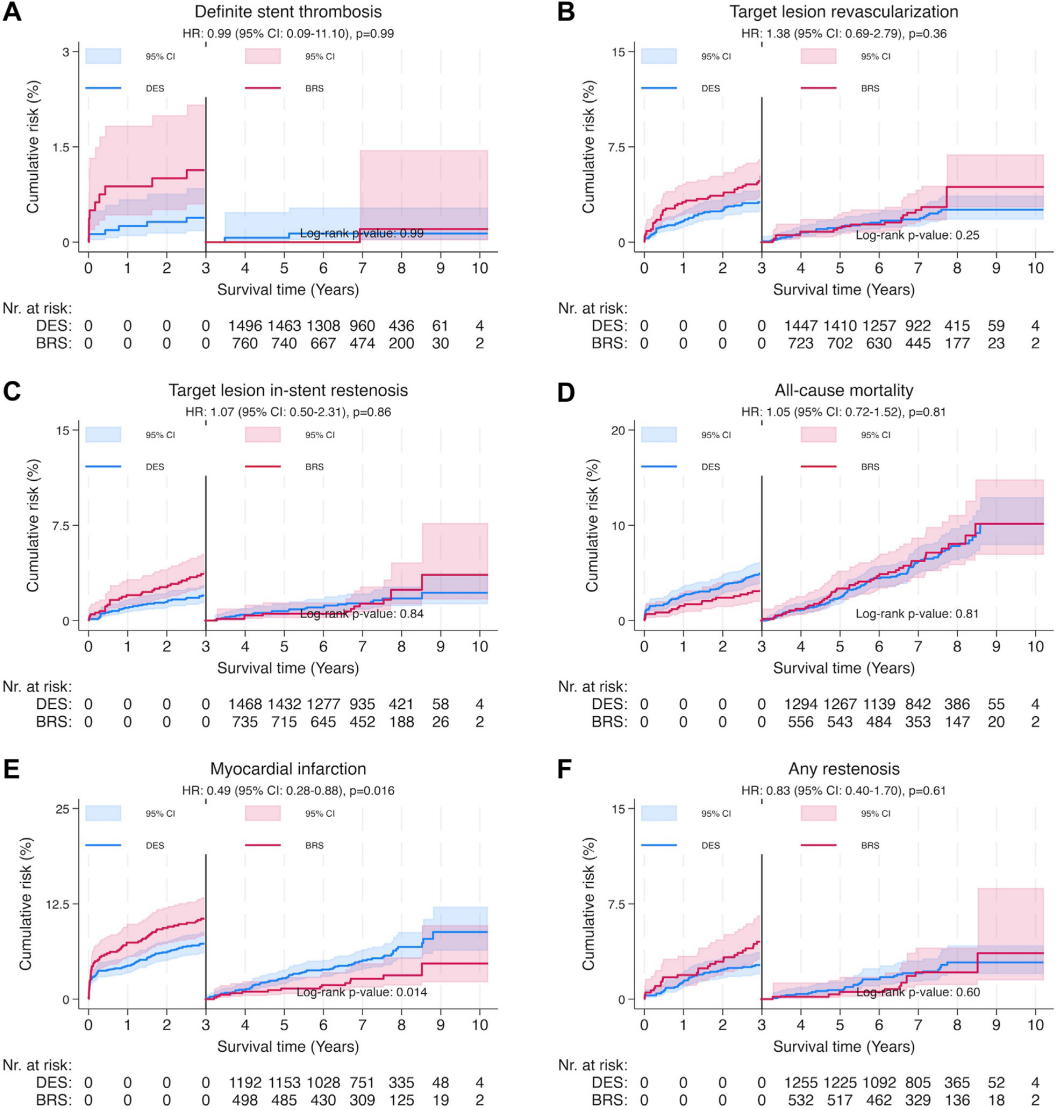
**Landmark analysis.** Kaplan–Meier curves, outcome. Panels **A**-**F** show Kaplan-Meier event curves for ST, TLR, ISR, all-cause mortality, MI, and any restenosis respectively. BRS, Absorb everolimus-eluting bioresorbable scaffold; DES, drug-eluting stent; HR, hazard ratio.

Kaplan–Meier curves for landmark analyses after 3 years are displayed in Figure 3. No significant differences were noted for ST, TLR, ISR, all-cause mortality, or any restenosis (*P* > .05). The CI for ST was wide (0.09-11.10), the numerically higher event rate of ISR with BRS did not reach statistical significance (*P* = .86), and long-term all-cause mortality was nearly identical between the 2 strategies. In contrast, the rate of MI was lower (HR, 0.49; 95% CI, 0.28-0.88; *P* = .016) for BRS than for DES.

### Subgroup analysis of ST and MI

**Figure 4:**
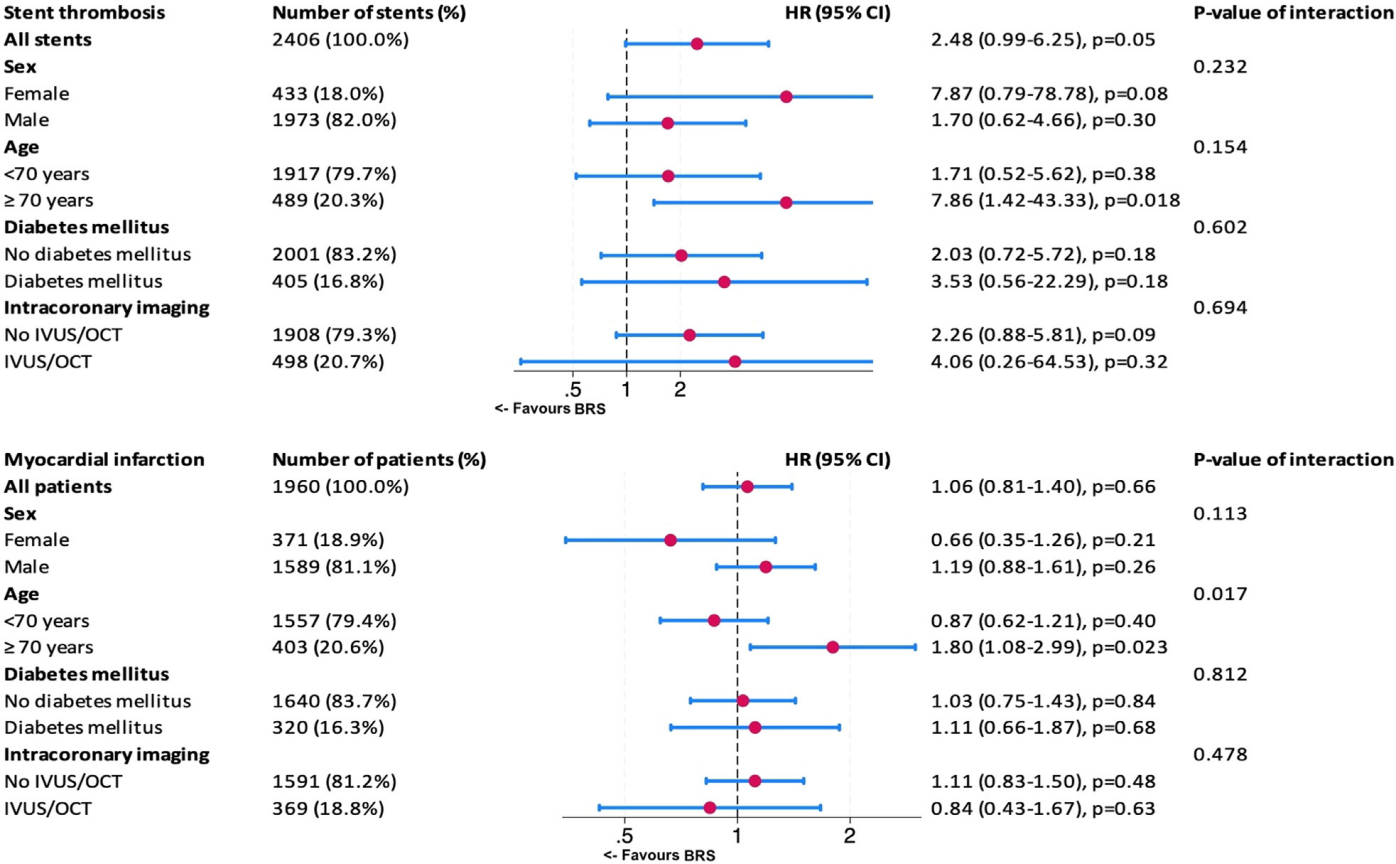
**Subgroup analysis.** Rates of stent thrombosis and myocardial infarction, Absorb everolimus-eluting bioresorbable scaffolds (BRS) vs drug-eluting stents. HR, hazard ratio; IVUS, intravascular ultrasound; OCT, optical coherence tomography.

The results of the subgroup analysis are displayed in Figure 4. For ST, there was an increased HR with BRS compared to DES (HR, 2.5; 95% CI, 0.99-6.25; *P* = .05). Both male and female patients exhibited higher HR with BRS, although CI were wide, and no statistically significant interaction by sex was observed. Similarly, no significant differences were found between the diabetes and nondiabetes subgroups. The use of intravascular imaging did not modify the relative risk for ST. Imaging use did not significantly alter the relative risk for ST. Among patients aged ≥70 years, the HR for ST with BRS was notably higher, although the interaction *P* value (P = .2) suggests a nonsignificant interaction.

The subgroup analysis for MI indicated no overall significant difference between BRS and DES in the studied population. Similarly, there was no statistically significant interaction for sex, diabetes mellitus, or the use of intravascular imaging. However, patients ≥70 years had a significantly higher HR for MI with BRS compared to DES (*P* = .02).

## Discussion

In this large, real-world population study, all-cause mortality and MI rates with BRS were comparable to those observed with DES. However, BRS was associated with higher rates of ST, TLR, and ISR, which persisted throughout the study period. Notably, a landmark analysis beyond 3 years demonstrated a late convergence with BRS showing reduced MI rates during extended follow-up (Central Illustration).

**Central Illustration:**
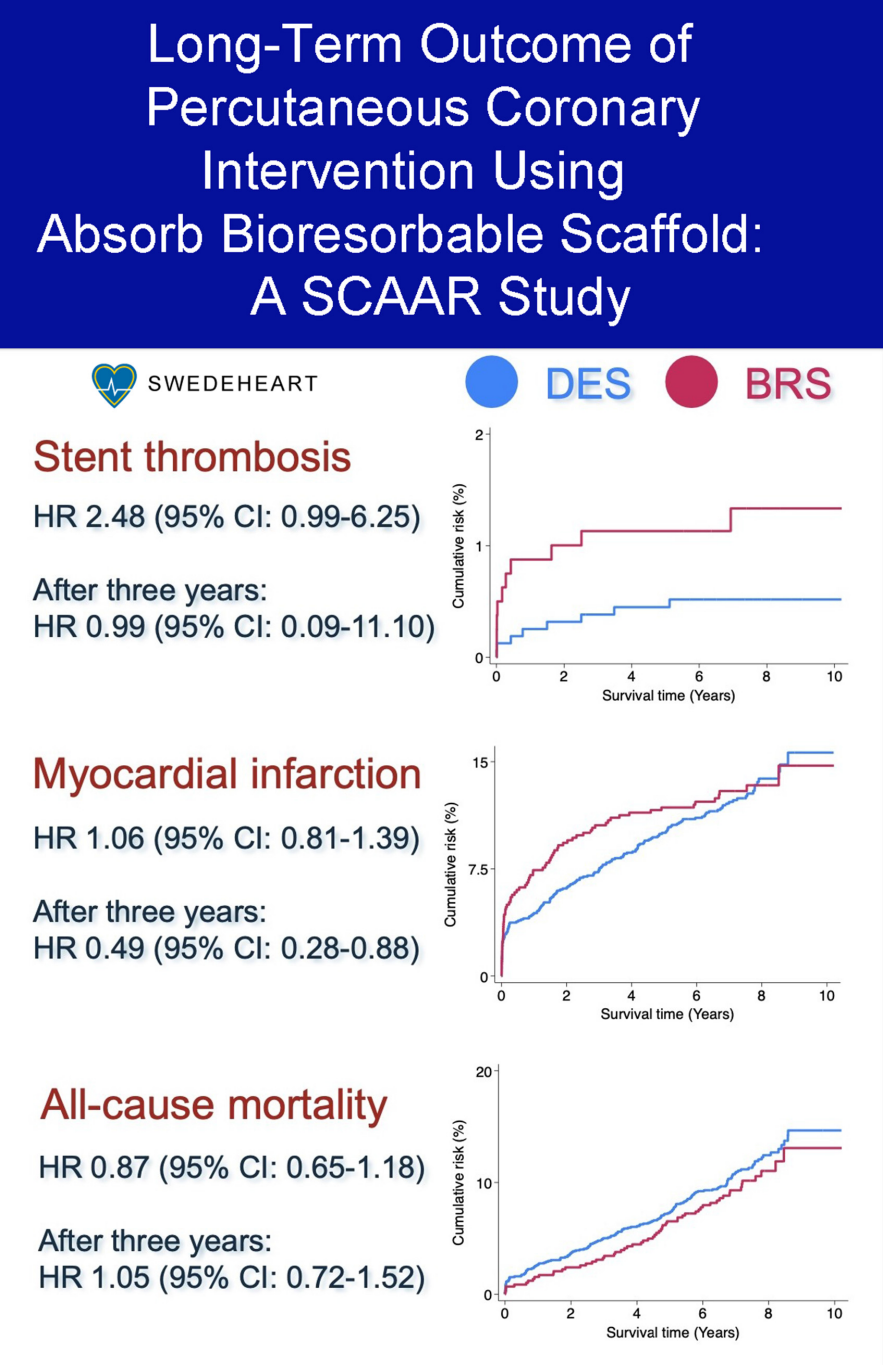
Long-term outcome of percutaneous coronary intervention (PCI) using Absorb bioresorbable scaffold: a SCAAR study. BRS, bioresorbable scaffolds; DES, drug-eluting stents; HR, hazard ratio.

During the full study period, similar to 2 different meta-analyses by Stone et al^7^ and Ali et al^8^ and the implantation technique-optimized (optimal lesion preparation as well as routine scaffolding pre- and postdilatation) ABSORB IV,^3^ which somewhat improved early outcomes, there were no differences in all-cause mortality between BRS and DES. Despite early device-related complications, also displayed in other trials,^3^^,^^7^, ^8^, ^9^ long-term survival did not appear to be adversely affected by the choice of scaffolding. This likely reflects the multifactorial nature of mortality in chronic coronary disease, where device-related events represent only one of many potential causes of death. Similarly, the cumulative risk of MI between the 2 groups did not differ significantly, which is in contrast to previous reports^3^^,^^7^^,^^8^; however, our findings in the landmark analysis offers an explanation. The higher early rate of ST did not translate into an increased overall MI burden. This may be due to the relatively small number of events and the fact that many other processes beyond stented segments may drive MI risk in long-term follow-up, as the risk of recurrent MI is twice as high for lesions in previously untreated arteries than in those that had been stented.^10^ While the Kaplan–Meyer any restenosis event rate for the BRS was higher than that of DES, the observed difference did not reach statistical significance. One may argue that there may not have been enough events to detect small differences, and it is plausible that the event-driven data, coupled with the absence of routine angiographic follow-up in asymptomatic patients, may have led to underdetection of certain events. Any restenosis appears to be predominantly driven by vascular healing responses to scaffolding placement. Hence, similar any restenosis rates between devices could be explained by device placement itself rather than type of device used. Our findings of increased rates of ST, TLR, and ISR with BRS compared with contemporary DES are consistent with previous randomized controlled trials, such as ABSORB II and ABSORB IV, 2 meta-analyses,^7^^,^^8^ and real-world data from a previous SCAAR registry report.^2^^,^^3^^,^^9^

In the landmark analysis starting at 3 years, the rates of ST were similar for BRS and DES, which is consistent with previous findings.^3^^,^^7^ The wide CI for ST reflects low event rates and limited power to detect meaningful differences, but overall, both devices appear comparable in their late safety profile. There was no difference in ISR and TLR between groups, suggesting that the bioresorbable scaffold’s theoretical advantage “leave nothing behind” and vessel restoration does not translate into lower adverse device-related outcomes compared to DES, possibly due to incomplete scaffold resorption or lack of radial support.

There was no survival or any restenosis difference between strategies, consistent with evidence from the ABSORB IV randomized controlled trial.^3^ However, in contrast to previous studies,^3^^,^^7^ the significant reduction in MI with BRS from 3 years onward is a striking finding, suggesting a potential long-term benefit over DES once the scaffold is resorbed and the vessel may have returned to a more physiological state. Notably, the ABSORB IV 5-year data did not include a separate landmark analysis of recurrent MI only but of target vessel MI (which did not show a significant difference between BRS and DES beyond 3 years). Kaplan–Meier curves indicated a relatively uniform distribution over time and linear accumulation of MI for both BRS and DES groups, suggesting similar long-term risk patterns. Continuous reporting from the major Absorb trials will shed more light on this matter. Additionally, early data and ongoing trials with alternative resorbable devices provide a basis for cautious optimism, indicating sustained interest and potential for improvement in resorbable device technology.^11^^,^^12^

Our findings allow us to speculate that uncaging and the restoration of vasomotor function, at least in part, could explain the lower rates of MI in the long run, compared with caged vessels and DES. A recent randomized control trial investigating a novel bioadaptor device with restoration of vasomotor function showed lower rates of TLR and target vessel revascularization from 6 months to 1 year, compared with a modern DES. This coincides with bioadaptor uncaging, suggesting that this mechanism may be of clinical importance.^13^ Another potential explanation for the lower MI rates with BRS may be the differences in dual antiplatelet therapy duration, since the fear of late ST with BRS may have caused clinicians to extend dual antiplatelet therapy courses for this subset of patients. Unfortunately, in the current study, we do not have data on the duration of dual antiplatelet therapy after any stent implantation.

Although BRS were introduced with the aim to “leave nothing behind” and potentially lower very-late adverse events associated with permanent metallic stents, these long-term data suggest a trade-off involving higher initial complication rates vs potential long-term benefits. The BRS are associated with higher overall rates of ST, TLR, and ISR compared with contemporary DES. However, from 3 years onwards, these outcomes occurred at similar rates, while BRS displayed lower rates of MI compared with DES. It may be hypothesized that there is a time point after implantation where the advantages of BRS outweigh the early shortcomings, possibly by restoring some aspects of normal vascular physiology and reducing the long-term atherothrombotic risk. This may eventually lead to net superior clinical outcomes with even more extended follow-up and comprehensive longitudinal studies.

In the subgroup analysis for ST, all stents showed a consistent increased risk with BRS compared to DES, suggesting that, in a broad population, BRS may carry a somewhat higher risk of thrombosis. There was no statistically significant interaction with sex, and diabetes status did not clearly alter the relative performance of BRS. Patients ≥70 years showed a notably higher hazard ratio for ST with BRS. While the interaction *P* value does not firmly confirm age as a statistically significant modifier, the numerical difference may be clinically concerning. Similar to the analysis for ST, the subgroup analysis for MI showed no meaningful interaction for sex, diabetes mellitus, or the use of intravascular imaging. However, in contrast to interactions for ST, the age effect was more pronounced and statistically significant for MI. This suggests that older patients may be harmed by the use of BRS, potentially reflecting issues such as more complex coronary anatomy, more challenging scaffold deployment, slower and less predictable device resorption, or potentially shorter exposure to dual antiplatelet therapy in older, often more comorbid patients. Considering the reduced life expectancy associated with advancing age, it is plausible to infer that patients aged ≥70 years are more likely to endure the initial adverse effects of BRS but may not survive sufficiently long to gain from its possible long-term benefits.

### Limitations

All recorded events were clinically driven and there was no active safety surveillance, therefore asymptomatic outcome events may have escaped notice. TLR-, ST-, and ISR-events were operator-reported events and nonadjudicated/core-lab evaluated, although regular assessments were completed on a subset of patients to ensure source data adherence and reporting. Previous large monitoring has shown a reporting data consistency of 97% between sites (unpublished). Despite the use of PS matching, there is potential for residual confounding such that variables not captured by the study still influenced outcomes. Moreover, outcomes in the landmark analysis were conditional upon patients remaining event-free up to 3 years. The lack of data on the duration of dual antiplatelet therapy restricts our ability to fully assess the impact of antiplatelet treatment on long-term clinical outcomes.

## Conclusion

In this large, real-world population study, BRS showed early inferior clinical performance compared with contemporary DES. Beyond 3 years, device-related outcomes were similar while MI rates favored BRS. These findings suggest a complex trade-off between early device-related events and potential long-term benefits of BRS-mediated vascular restoration.

## Declaration of competing interest

The authors declared no potential conflicts of interest with respect to the research, authorship, and/or publication of this article.

## Funding sources

This work was partly funded by Region Vastmanland Centre for Clinical Research (SS), Swedish Scientific Research Council, Heartlung Foundation (SvK), and SUS Stiftelser och Fonder (trusts and funds) (SK).

## Ethics statement and patient consent

The report adhered to the relevant ethical guidelines and was approved by the Swedish Ethical Review Authority with diary number 2021-04940.

## Figures and Tables

**Supplemental Table S1:**