# Supplementary material for: Long-Term Outcome of Percutaneous Coronary Intervention Using Absorb Bioresorbable Scaffold: A SCAAR Study
Source: J Soc Cardiovasc Angiogr Interv. 2025 Jul 29;4(9):103724. doi: 10.1016/j.jscai.2025.103724 (PMC12485528; doi:10.1016/j.jscai.2025.103724)
Supplement: Supplemental Tables [file mmc1.docx]

**Supplements**

**Supplementary Table S1**. Baseline characteristics before PS matching

| **Patient-level** |  | **DES** | **Absorb BRS** | **p-value** | **Missing** |
| --- | --- | --- | --- | --- | --- |
|  |  | **(n=85203)** | **(n=598)** |  | **(%)** |
| **Patient demographics** |  |  |  |  |  |
| Inclusion time | 2011-2013 | 25146 (29.5%) | 168 (28.1%) | <0.001 | 0.0 |
|  | 2014 | 13989 (16.4%) | 222 (37.1%) |  |  |
|  | 2015-2018 | 46068 (54.1%) | 208 (34.8%) |  |  |
| Age, mean (SD) |  | 67.9 (10.9) | 60.2 (11.3) | <0.001 | 0.0 |
| Age ≥ 80 |  | 12927 (15.2%) | 25 (4.2%) | <0.001 | 0.0 |
| Sex | Men | 63218 (74.2%) | 481 (80.4%) | <0.001 | 0.0 |
| Smoking status | Non-smoker | 33284 (41.1%) | 250 (43.3%) | 0.14 | 5.0 |
|  | Previous smoker | 31871 (39.4%) | 204 (35.4%) |  |  |
|  | Active smoker | 15792 (19.5%) | 123 (21.3%) |  |  |
| **Comorbidities** |  |  |  |  |  |
| Diabetes Mellitus |  | 19486 (22.9%) | 102 (17.1%) | <0.001 | 0.0 |
| Hypertension |  | 29563 (34.7%) | 135 (22.6%) | <0.001 | 0.0 |
| Hyperlipidaemia |  | 41402 (49.3%) | 261 (44.1%) | 0.012 | 1.4 |
| Heart failure |  | 6045 (7.1%) | 17 (2.8%) | <0.001 | 0.0 |
| Renal failure |  | 2783 (3.3%) | 6 (1.0%) | 0.002 | 0.0 |
| Estimated GFR (ml/min/1,73 m²), mean (SD) |  | 82.1 (24.4) | 88.2 (22.7) | <0.001 | 27.1 |
| Previous stroke |  | 5274 (6.2%) | 19 (3.2%) | 0.002 | 0.0 |
| Previous MI |  | 15239 (17.9%) | 81 (13.5%) | 0.006 | 0.0 |
| Previous PCI |  | 14521 (17.0%) | 90 (15.1%) | 0.20 | 0.0 |
| Previous CABG |  | 6483 (7.6%) | 12 (2.0%) | <0.001 | 0.0 |
| **Procedural characteristics** |  |  |  |  |  |
| Indication | CCD | 17502 (20.5%) | 161 (26.9%) | <0.001 | 0.0 |
|  | Unstable angina | 19660 (23.1%) | 120 (20.1%) |  |  |
|  | Non-STEMI | 20662 (24.3%) | 176 (29.4%) |  |  |
|  | STEMI | 23795 (27.9%) | 117 (19.6%) |  |  |
|  | Other | 3584 (4.2%) | 24 (4.0%) |  |  |
| Contrast volume (ml), mean (SD) |  | 161.4 (74.9) | 150.4 (73.0) | <0.001 | 0.0 |
| Arterial access | Radial | 68816 (80.8%) | 553 (92.5%) | <0.001 | 0.0 |
|  | Femoral | 15157 (17.8%) | 44 (7.4%) |  |  |
|  | Other | 1230 (1.4%) | 1 (0.2%) |  |  |
| Use of intracoronary imaging |  | 3972 (4.7%) | 118 (19.7%) | <0.001 | 0.0 |
| Angiographic findings | 1 vessel disease | 39422 (46.5%) | 393 (66.2%) | <0.001 | 0.5 |
|  | 2 vessel disease | 25387 (29.9%) | 146 (24.6%) |  |  |
|  | 3 vessel disease and/or left main disease | 19970 (23.6%) | 55 (9.3%) |  |  |
| Number of stents, mean (SD) |  | 1.75 (1.06) | 1.75 (1.15) | 0.879 |  |
| Complete revascularization |  | 57539 (68.4%) | 524 (88.1%) | <0.001 | 1.3 |
| **Medications prior to PCI** |  |  |  |  |  |
| ASA |  | 29869 (35.1%) | 208 (34.8%) | 0.89 | 0.0 |
| P2Y12 |  | 6472 (7.6%) | 63 (10.5%) | 0.007 | 0.0 |
| Statin |  | 29100 (34.2%) | 206 (34.4%) | 0.88 | 0.0 |
| Beta-blocker |  | 30155 (35.4%) | 193 (32.3%) | 0.11 | 0.0 |
| ARB/ACEi |  | 27632 (32.4%) | 165 (27.6%) | 0.012 | 0.0 |
| Calcium channel blocker |  | 16840 (19.8%) | 75 (12.5%) | <0.001 | 0.0 |
| **Stent-level** |  | **DES** | **Absorb BRS** | **p-value** |  |
|  |  | **(n=139488)** | **(n=820)** |  |  |
| **Lesion characteristics** |  |  |  |  |  |
| Lesion location | Left main lesion | 5366 (3.8%) | 5 (0.6%) | <0.001 | 0.0 |
|  | Other proximal lesion | 55071 (39.5%) | 292 (35.6%) |  |  |
|  | Distal lesion | 79051 (56.7%) | 523 (63.8%) |  |  |
| Lesion classification | Type A | 12056 (8.6%) | 74 (9.0%) | 0.002 | 0.0 |
|  | Type B1-B2 | 83307 (59.7%) | 537 (65.5%) |  |  |
|  | Type C or B1-B2 with bifurcation | 44020 (31.6%) | 208 (25.4%) |  |  |
|  | Other | 105 (0.1%) | 1 (0.1%) |  |  |
| **Stent characteristics** |  |  |  |  |  |
| Stent diameter (mm), mean (SD) |  | 3.1 (0.5) | 3.3 (0.4) | <0.001 | 0.0 |
| Stent length (mm), mean (SD) |  | 21.3 (8.4) | 19.9 (6.1) | <0.001 | 0.0 |
| Use of post dilation |  | 54194 (38.9%) | 526 (64.1%) | <0.001 | 0.0 |
| Stent name | Biosensors BioMatrix | 2112 (1.5%) | 0 (0.0%) | <0.001 | 0.0 |
|  | Abbott Xience Prime | 5427 (3.9%) | 0 (0.0%) |  |  |
|  | BS Promus Element | 5981 (4.3%) | 0 (0.0%) |  |  |
|  | Medtronic Resolute Integrity | 18944 (13.6%) | 0 (0.0%) |  |  |
|  | Biotronik Orsiro | 8944 (6.4%) | 0 (0.0%) |  |  |
|  | Abbott ABSORB BVS | 0 (0.0%) | 820 (100.0%) |  |  |
|  | BS Promus Element Plus | 5572 (4.0%) | 0 (0.0%) |  |  |
|  | Abbott Xience Xpedition | 10955 (7.9%) | 0 (0.0%) |  |  |
|  | BS Promus Premier | 24994 (17.9%) | 0 (0.0%) |  |  |
|  | BS Synergy | 21862 (15.7%) | 0 (0.0%) |  |  |
|  | Terumo Ultimaster | 2658 (1.9%) | 0 (0.0%) |  |  |
|  | Biosensors Biofreedom | 1788 (1.3%) | 0 (0.0%) |  |  |
|  | Medtronic Resolute Onyx | 24690 (17.7%) | 0 (0.0%) |  |  |
|  | Abbott Xience ProX | 4470 (3.2%) | 0 (0.0%) |  |  |
|  | Abbott Xience Alpine | 1091 (0.8%) | 0 (0.0%) |  |  |

Footnote: Categorical variables: Count (Percentage), n: Number. BRS: Absorb everolimus-eluting bioresorbable scaffolds; CABG: Coronary artery bypass graft surgery; CCD: Chronic coronary disease; DES: Drug-eluting stents; GFR: Glomerular filtration rate; MI: myocardial infarction; PCI: Percutaneous coronary intervention; STEMI: ST-elevation MI.

Abbott Xience family: Abbott group, Biosensors family: Biosensors Interventional Technologies Pte Ltd, Biotronic Orsiro: Biotronic group, BS family: Boston scientific (BS) corporation, Medtronic Resolute family: Medtronic PLC, Terumo Ultimaster: Terumo corporation.

**Supplementary Figure S1**

Supplementary_figure1.jpg

Footnote: **Standardized % bias across covariates**. *X-axis: Absolute standardised mean difference. Y-axis: Covariates. "All" (open circles): Standardised differences before matching. "Matched" (solid dots): Standardised differences after applying matching.*

**Supplementary Table S2 A**. Baseline characteristics of patient eligible for “Definite stent thrombosis” landmark analysis

| **Patient-level** |  | **DES** | **Absorb BRS** | **p-value** |
| --- | --- | --- | --- | --- |
|  |  | **(n=1303)** | **(n=558)** |  |
| **Patient demographics** |  |  |  |  |
| Inclusion time | 2011-2013 | 382 (29.3%) | 151 (27.1%) | 0.42 |
|  | 2014 | 497 (38.1%) | 209 (37.5%) |  |
|  | 2015-2018 | 424 (32.5%) | 198 (35.5%) |  |
| Age, mean (SD) |  | 59.7 (10.9) | 59.8 (11.0) | 0.91 |
| Age ≥ 80 |  | 46 (3.5%) | 17 (3.0%) | 0.60 |
| Sex | Men | 1065 (81.7%) | 449 (80.5%) | 0.52 |
| Smoking status | Non-smoker | 535 (42.1%) | 235 (43.4%) | 0.23 |
|  | Previous smoker | 423 (33.3%) | 193 (35.6%) |  |
|  | Active smoker | 314 (24.7%) | 114 (21.0%) |  |
| **Comorbidities** |  |  |  |  |
| Diabetes Mellitus |  | 203 (15.6%) | 92 (16.5%) | 0.62 |
| Hypertension |  | 260 (20.0%) | 121 (21.7%) | 0.40 |
| Hyperlipidaemia |  | 535 (41.1%) | 245 (43.9%) | 0.25 |
| Heart failure |  | 23 (1.8%) | 12 (2.2%) | 0.57 |
| Renal failure |  | 6 (0.5%) | 5 (0.9%) | 0.26 |
| Estimated GFR, (ml/min/1,73 m²), mean (SD) |  | 89.0 (20.8) | 88.9 (22.3) | 0.99 |
| Previous stroke |  | 38 (2.9%) | 17 (3.0%) | 0.88 |
| Previous MI |  | 141 (10.8%) | 76 (13.6%) | 0.085 |
| Previous PCI |  | 168 (12.9%) | 85 (15.2%) | 0.18 |
| Previous CABG |  | 23 (1.8%) | 10 (1.8%) | 0.97 |
| **Procedural characteristics** |  | 203 (15.6%) | 92 (16.5%) | 0.62 |
| Indication | Stable CAD | 364 (27.9%) | 153 (27.4%) | 0.98 |
|  | Unstable angina | 268 (20.6%) | 113 (20.3%) |  |
|  | Non-STEMI | 367 (28.2%) | 165 (29.6%) |  |
|  | STEMI | 252 (19.3%) | 106 (19.0%) |  |
|  | Other | 52 (4.0%) | 21 (3.8%) |  |
| Contrast volume (ml), mean (SD) |  | 168.7 (77.4) | 150.4 (73.0) | <0.001 |
| Arterial access | Radial | 1229 (94.3%) | 516 (92.5%) | 0.28 |
|  | Femoral | 71 (5.4%) | 41 (7.3%) |  |
|  | Other | 3 (0.2%) | 1 (0.2%) |  |
| Use of intracoronary imaging |  | 238 (18.3%) | 106 (19.0%) | 0.71 |
| Angiographic findings | 1 vessel disease | 854 (65.5%) | 372 (66.7%) | 0.77 |
|  | 2 vessel disease | 340 (26.1%) | 137 (24.6%) |  |
|  | 3 vessel disease and/or left main disease | 109 (8.4%) | 49 (8.8%) |  |
| Number of stents, mean (SD) |  | 1.9 (1.1) | 1.8 (1.2) | 0.065 |
| Complete revascularization |  | 1132 (86.9%) | 492 (88.2%) | 0.44 |
| **Medications prior to PCI** |  |  |  |  |
| ASA |  | 420 (32.2%) | 193 (34.6%) | 0.32 |
| P2Y12 |  | 142 (10.9%) | 59 (10.6%) | 0.84 |
| Statin |  | 409 (31.4%) | 196 (35.1%) | 0.11 |
| Beta-blocker |  | 368 (28.2%) | 180 (32.3%) | 0.082 |
| ARB/ACEi |  | 349 (26.8%) | 155 (27.8%) | 0.66 |
| Calcium channel blocker |  | 183 (14.0%) | 70 (12.5%) | 0.39 |
| **Stent-level** |  | **DES** | **Absorb BRS** | **p-value** |
|  |  | **(n=1517)** | **(n=770)** |  |
| **Lesion characteristics** |  |  |  |  |
| Lesion location | Left main lesion | 6 (0.4%) | 3 (0.4%) | 1.00 |
|  | Other proximal lesion | 533 (35.1%) | 272 (35.3%) |  |
|  | Distal lesion | 978 (64.5%) | 495 (64.3%) |  |
| Lesion classification | Type A | 133 (8.8%) | 72 (9.4%) | 0.86 |
|  | Type B1-B2 | 990 (65.3%) | 507 (65.8%) |  |
|  | Type C or B1-B2 with bifurcation | 393 (25.9%) | 190 (24.7%) |  |
|  | Other | 1 (0.1%) | 1 (0.1%) |  |
| **Stent characteristics** |  |  |  |  |
| Stent diameter (mm), mean (SD) |  | 3.1 (0.5) | 3.2 (0.4) | <0.001 |
| Stent length (mm), mean (SD) |  | 20.7 (8.0) | 19.9 (6.1) | 0.011 |
| Use of postdilation |  | 547 (36.1%) | 496 (64.4%) | <0.001 |
| Stent name | Biosensors BioMatrix | 50 (3.3%) | 0 (0.0%) | <0.001 |
|  | Abbott Xience Prime | 14 (0.9%) | 0 (0.0%) |  |
|  | BS Promus Element | 22 (1.5%) | 0 (0.0%) |  |
|  | Medtronic Resolute Integrity | 353 (23.3%) | 0 (0.0%) |  |
|  | Biotronik Orsiro | 98 (6.5%) | 0 (0.0%) |  |
|  | Abbott ABSORB BVS | 0 (0.0%) | 770 (100.0%) |  |
|  | BS Promus Element Plus | 58 (3.8%) | 0 (0.0%) |  |
|  | Abbott Xience Xpedition | 153 (10.1%) | 0 (0.0%) |  |
|  | BS Promus Premier | 376 (24.8%) | 0 (0.0%) |  |
|  | BS Synergy | 178 (11.7%) | 0 (0.0%) |  |
|  | Terumo Ultimaster | 36 (2.4%) | 0 (0.0%) |  |
|  | Biosensors Biofreedom | 3 (0.2%) | 0 (0.0%) |  |
|  | Medtronic Resolute Onyx | 168 (11.1%) | 0 (0.0%) |  |
|  | Abbott Xience ProX | 8 (0.5%) | 0 (0.0%) |  |
|  |  |  |  |  |

Footnote: Categorical variables: Count(Percentage).

Abbott Xience family: Abbott group, Biosensors family: Biosensors Interventional Technologies Pte Ltd, Biotronic Orsiro: Biotronic group, BS family: Boston scientific (BS) corporation, Medtronic Resolute family: Medtronic PLC, Terumo Ultimaster: Terumo corporation.

**Supplementary Table S2 B**. Baseline characteristics of patient eligible for “Target lesion revascularization” landmark analysis

| **Patient-level** |  | **DES** | **Absorb BRS** | **p-value** |
| --- | --- | --- | --- | --- |
|  |  | **(n=1273)** | **(n=541)** |  |
| **Patient demographics** |  |  |  |  |
| Inclusion time | 2011-2013 | 373 (29.3%) | 143 (26.4%) | 0.28 |
|  | 2014 | 486 (38.2%) | 203 (37.5%) |  |
|  | 2015-2018 | 414 (32.5%) | 195 (36.0%) |  |
| Age, mean (SD) |  | 59.7 (10.9) | 59.8 (11.0) | 0.82 |
| Age ≥ 80 |  | 43 (3.4%) | 16 (3.0%) | 0.64 |
| Sex | Men | 1043 (81.9%) | 436 (80.6%) | 0.50 |
| Smoking status | Non-smoker | 521 (41.9%) | 225 (42.9%) | 0.18 |
|  | Previous smoker | 415 (33.4%) | 191 (36.4%) |  |
|  | Active smoker | 307 (24.7%) | 109 (20.8%) |  |
| **Comorbidities** |  |  |  |  |
| Diabetes Mellitus |  | 194 (15.2%) | 86 (15.9%) | 0.72 |
| Hypertension |  | 248 (19.5%) | 118 (21.8%) | 0.26 |
| Hyperlipidaemia |  | 519 (40.8%) | 236 (43.6%) | 0.26 |
| Heart failure |  | 21 (1.6%) | 12 (2.2%) | 0.41 |
| Renal failure |  | 6 (0.5%) | 5 (0.9%) | 0.26 |
| Estimated GFR, (ml/min/1,73 m²), mean (SD) |  | 89.0 (20.9) | 89.0 (22.3) | 0.98 |
| Previous stroke |  | 36 (2.8%) | 17 (3.1%) | 0.72 |
| Previous MI |  | 136 (10.7%) | 71 (13.1%) | 0.13 |
| Previous PCI |  | 162 (12.7%) | 79 (14.6%) | 0.28 |
| Previous CABG |  | 22 (1.7%) | 8 (1.5%) | 0.70 |
| **Procedural characteristics** |  |  |  |  |
| Indication | Stable CAD | 354 (27.8%) | 147 (27.2%) | 0.99 |
|  | Unstable angina | 257 (20.2%) | 112 (20.7%) |  |
|  | Non-STEMI | 362 (28.4%) | 158 (29.2%) |  |
|  | STEMI | 248 (19.5%) | 103 (19.0%) |  |
|  | Other | 52 (4.1%) | 21 (3.9%) |  |
| Contrast volume (ml), mean (SD) |  | 168.8 (77.8) | 149.5 (71.3) | <0.001 |
| Arterial access | Radial | 1201 (94.3%) | 501 (92.6%) | 0.33 |
|  | Femoral | 69 (5.4%) | 39 (7.2%) |  |
|  | Other | 3 (0.2%) | 1 (0.2%) |  |
| Use of intracoronary imaging |  | 231 (18.1%) | 103 (19.0%) | 0.65 |
| Angiographic findings | 1 vessel disease | 836 (65.7%) | 363 (67.1%) | 0.72 |
|  | 2 vessel disease | 331 (26.0%) | 131 (24.2%) |  |
|  | 3 vessel disease and/or left main disease | 106 (8.3%) | 47 (8.7%) |  |
| Number of stents, mean (SD) |  | 1.9 (1.1) | 1.8 (1.2) | 0.081 |
| Complete revascularization |  | 1108 (87.0%) | 482 (89.1%) | 0.22 |
| **Medications prior to PCI** |  |  |  |  |
| ASA |  | 405 (31.8%) | 185 (34.2%) | 0.32 |
| P2Y12 |  | 138 (10.8%) | 54 (10.0%) | 0.59 |
| Statin |  | 397 (31.2%) | 188 (34.8%) | 0.14 |
| Beta-blocker |  | 356 (28.0%) | 173 (32.0%) | 0.085 |
| ARB/ACEi |  | 335 (26.3%) | 148 (27.4%) | 0.65 |
| Calcium channel blocker |  | 177 (13.9%) | 68 (12.6%) | 0.45 |
| **Stent-level** |  | **DES** | **Absorb BRS** | **p-value** |
|  |  | **(n=1477)** | **(n=738)** |  |
| **Lesion characteristics** |  |  |  |  |
| Lesion location | Left main lesion | 6 (0.4%) | 3 (0.4%) | 1.00 |
|  | Other proximal lesion | 518 (35.1%) | 259 (35.1%) |  |
|  | Distal lesion | 953 (64.5%) | 476 (64.5%) |  |
| Lesion classification | Type A | 131 (8.9%) | 71 (9.6%) | 0.81 |
|  | Type B1-B2 | 964 (65.3%) | 486 (65.9%) |  |
|  | Type C or B1-B2 with bifurcation | 381 (25.8%) | 180 (24.4%) |  |
|  | Other | 1 (0.1%) | 1 (0.1%) |  |
| **Stent characteristics** |  |  |  |  |
| Stent diameter (mm), mean (SD) |  | 3.1 (0.5) | 3.3 (0.4) | <0.001 |
| Stent length (mm), mean (SD) |  | 20.8 (8.0) | 19.8 (6.0) | 0.004 |
| Use of postdilation |  | 525 (35.5%) | 471 (63.8%) | <0.001 |
| Stent name | Biosensors BioMatrix | 47 (3.2%) | 0 (0.0%) | <0.001 |
|  | Abbott Xience Prime | 14 (0.9%) | 0 (0.0%) |  |
|  | BS Promus Element | 21 (1.4%) | 0 (0.0%) |  |
|  | Medtronic Resolute Integrity | 341 (23.1%) | 0 (0.0%) |  |
|  | Biotronik Orsiro | 96 (6.5%) | 0 (0.0%) |  |
|  | Abbott ABSORB BVS | 0 (0.0%) | 738 (100.0%) |  |
|  | BS Promus Element Plus | 57 (3.9%) | 0 (0.0%) |  |
|  | Abbott Xience Xpedition | 149 (10.1%) | 0 (0.0%) |  |
|  | BS Promus Premier | 372 (25.2%) | 0 (0.0%) |  |
|  | BS Synergy | 175 (11.8%) | 0 (0.0%) |  |
|  | Terumo Ultimaster | 34 (2.3%) | 0 (0.0%) |  |
|  | Biosensors Biofreedom | 3 (0.2%) | 0 (0.0%) |  |
|  | Medtronic Resolute Onyx | 160 (10.8%) | 0 (0.0%) |  |
|  | Abbott Xience ProX | 8 (0.5%) | 0 (0.0%) |  |
|  |  |  |  |  |

Footnote: Categorical variables: Count(Percentage).

Abbott Xience family: Abbott group, Biosensors family: Biosensors Interventional Technologies Pte Ltd, Biotronic Orsiro: Biotronic group, BS family: Boston scientific (BS) corporation, Medtronic Resolute family: Medtronic PLC, Terumo Ultimaster: Terumo corporation.

**Supplementary Table S2 C**. Baseline characteristics of patient eligible for “Target lesion in-stent restenosis” landmark analysis

| **Patient-level** |  | **DES** | **Absorb BRS** | **p-value** |
| --- | --- | --- | --- | --- |
|  |  | **(n=1288)** | **(n=545)** |  |
| **Patient demographics** |  |  |  |  |
| Inclusion time | 2011-2013 | 375 (29.1%) | 145 (26.6%) | 0.34 |
|  | 2014 | 492 (38.2%) | 204 (37.4%) |  |
|  | 2015-2018 | 421 (32.7%) | 196 (36.0%) |  |
| Age, mean (SD) |  | 59.7 (10.9) | 59.8 (11.0) | 0.81 |
| Age ≥ 80 |  | 44 (3.4%) | 16 (2.9%) | 0.60 |
| Sex | Men | 1053 (81.8%) | 439 (80.6%) | 0.54 |
| Smoking status | Non-smoker | 527 (41.9%) | 227 (42.9%) | 0.24 |
|  | Previous smoker | 419 (33.3%) | 190 (35.9%) |  |
|  | Active smoker | 311 (24.7%) | 112 (21.2%) |  |
| **Comorbidities** |  |  |  |  |
| Diabetes Mellitus |  | 198 (15.4%) | 89 (16.3%) | 0.61 |
| Hypertension |  | 254 (19.7%) | 119 (21.8%) | 0.30 |
| Hyperlipidaemia |  | 528 (41.0%) | 238 (43.7%) | 0.29 |
| Heart failure |  | 23 (1.8%) | 12 (2.2%) | 0.55 |
| Renal failure |  | 6 (0.5%) | 5 (0.9%) | 0.25 |
| Estimated GFR, (ml/min/1,73 m²), mean (SD) |  | 88.9 (20.8) | 89.0 (22.4) | 0.97 |
| Previous stroke |  | 37 (2.9%) | 17 (3.1%) | 0.78 |
| Previous MI |  | 138 (10.7%) | 71 (13.0%) | 0.15 |
| Previous PCI |  | 164 (12.7%) | 80 (14.7%) | 0.26 |
| Previous CABG |  | 22 (1.7%) | 8 (1.5%) | 0.71 |
| **Procedural characteristics** |  |  |  |  |
| Indication | Stable CAD | 359 (27.9%) | 147 (27.0%) | 0.99 |
|  | Unstable angina | 261 (20.3%) | 113 (20.7%) |  |
|  | Non-STEMI | 366 (28.4%) | 160 (29.4%) |  |
|  | STEMI | 250 (19.4%) | 104 (19.1%) |  |
|  | Other | 52 (4.0%) | 21 (3.9%) |  |
| Contrast volume (ml), mean (SD) |  | 168.8 (77.7) | 149.4 (71.1) | <0.001 |
| Arterial access | Radial | 1215 (94.3%) | 505 (92.7%) | 0.36 |
|  | Femoral | 70 (5.4%) | 39 (7.2%) |  |
|  | Other | 3 (0.2%) | 1 (0.2%) |  |
| Use of intracoronary imaging |  | 235 (18.2%) | 105 (19.3%) | 0.61 |
| Angiographic findings | 1 vessel disease | 845 (65.6%) | 365 (67.0%) | 0.73 |
|  | 2 vessel disease | 337 (26.2%) | 133 (24.4%) |  |
|  | 3 vessel disease and/or left main disease | 106 (8.2%) | 47 (8.6%) |  |
| Number of stents, mean (SD) |  | 1.9 (1.1) | 1.8 (1.2) | 0.078 |
| Complete revascularization |  | 1122 (87.1%) | 483 (88.6%) | 0.37 |
| **Medications prior to PCI** |  |  |  |  |
| ASA |  | 412 (32.0%) | 186 (34.1%) | 0.37 |
| P2Y12 |  | 141 (10.9%) | 55 (10.1%) | 0.59 |
| Statin |  | 403 (31.3%) | 188 (34.5%) | 0.18 |
| Beta-blocker |  | 362 (28.1%) | 173 (31.7%) | 0.12 |
| ARB/ACEi |  | 342 (26.6%) | 150 (27.5%) | 0.67 |
| Calcium channel blocker |  | 178 (13.8%) | 69 (12.7%) | 0.51 |
| **Stent-level** |  | **DES** | **Absorb BRS** | **p-value** |
|  |  | **(n=1494)** | **(n=748)** |  |
| **Lesion characteristics** |  |  |  |  |
| Lesion location | Left main lesion | 6 (0.4%) | 3 (0.4%) | 1.00 |
|  | Other proximal lesion | 524 (35.1%) | 262 (35.0%) |  |
|  | Distal lesion | 964 (64.5%) | 483 (64.6%) |  |
| Lesion classification | Type A | 131 (8.8%) | 70 (9.4%) | 0.86 |
|  | Type B1-B2 | 974 (65.2%) | 492 (65.8%) |  |
|  | Type C or B1-B2 with bifurcation | 388 (26.0%) | 185 (24.7%) |  |
|  | Other | 1 (0.1%) | 1 (0.1%) |  |
| **Stent characteristics** |  |  |  |  |
| Stent diameter (mm), mean (SD) |  | 3.1 (0.5) | 3.3 (0.4) | <0.001 |
| Stent length (mm), mean (SD) |  | 20.8 (8.0) | 19.9 (6.0) | 0.006 |
| Use of postdilation |  | 532 (35.6%) | 478 (63.9%) | <0.001 |
| Stent name | Biosensors BioMatrix | 49 (3.3%) | 0 (0.0%) | <0.001 |
|  | Abbott Xience Prime | 14 (0.9%) | 0 (0.0%) |  |
|  | BS Promus Element | 21 (1.4%) | 0 (0.0%) |  |
|  | Medtronic Resolute Integrity | 346 (23.2%) | 0 (0.0%) |  |
|  | Biotronik Orsiro | 97 (6.5%) | 0 (0.0%) |  |
|  | Abbott ABSORB BVS | 0 (0.0%) | 748 (100.0%) |  |
|  | BS Promus Element Plus | 57 (3.8%) | 0 (0.0%) |  |
|  | Abbott Xience Xpedition | 151 (10.1%) | 0 (0.0%) |  |
|  | BS Promus Premier | 371 (24.8%) | 0 (0.0%) |  |
|  | BS Synergy | 176 (11.8%) | 0 (0.0%) |  |
|  | Terumo Ultimaster | 35 (2.3%) | 0 (0.0%) |  |
|  | Biosensors Biofreedom | 3 (0.2%) | 0 (0.0%) |  |
|  | Medtronic Resolute Onyx | 166 (11.1%) | 0 (0.0%) |  |
|  | Abbott Xience ProX | 8 (0.5%) | 0 (0.0%) |  |
|  |  |  |  |  |

Footnote: Categorical variables: Count(Percentage).

Abbott Xience family: Abbott group, Biosensors family: Biosensors Interventional Technologies Pte Ltd, Biotronic Orsiro: Biotronic group, BS family: Boston scientific (BS) corporation, Medtronic Resolute family: Medtronic PLC, Terumo Ultimaster: Terumo corporation.

**Supplementary Table S2 D**. Baseline characteristics of patient eligible for “All-cause mortality” landmark analysis

| **Patient-level** |  | **DES** | **Absorb BRS** | **p-value** |
| --- | --- | --- | --- | --- |
|  |  | **(n=1308)** | **(n=564)** |  |
| **Patient demographics** |  |  |  |  |
| Inclusion time | 2011-2013 | 384 (29.4%) | 154 (27.3%) | 0.48 |
|  | 2014 | 500 (38.2%) | 212 (37.6%) |  |
|  | 2015-2018 | 424 (32.4%) | 198 (35.1%) |  |
| Age, mean (SD) |  | 59.7 (10.9) | 59.9 (11.0) | 0.76 |
| Age ≥ 80 |  | 46 (3.5%) | 18 (3.2%) | 0.72 |
| Sex | Men | 1069 (81.7%) | 452 (80.1%) | 0.42 |
| Smoking status | Non-smoker | 535 (41.9%) | 236 (43.1%) | 0.21 |
|  | Previous smoker | 424 (33.2%) | 196 (35.8%) |  |
|  | Active smoker | 318 (24.9%) | 116 (21.2%) |  |
| **Comorbidities** |  |  |  |  |
| Diabetes Mellitus |  | 204 (15.6%) | 94 (16.7%) | 0.56 |
| Hypertension |  | 261 (20.0%) | 122 (21.6%) | 0.41 |
| Hyperlipidaemia |  | 537 (41.1%) | 248 (44.0%) | 0.24 |
| Heart failure |  | 23 (1.8%) | 12 (2.1%) | 0.59 |
| Renal failure |  | 6 (0.5%) | 5 (0.9%) | 0.27 |
| Estimated GFR, (ml/min/1,73 m²), mean (SD) |  | 89.0 (20.8) | 88.9 (22.2) | 0.92 |
| Previous stroke |  | 38 (2.9%) | 17 (3.0%) | 0.90 |
| Previous MI |  | 143 (10.9%) | 77 (13.7%) | 0.094 |
| Previous PCI |  | 170 (13.0%) | 86 (15.2%) | 0.19 |
| Previous CABG |  | 23 (1.8%) | 10 (1.8%) | 0.98 |
| **Procedural characteristics** |  |  |  |  |
| Indication | Stable CAD | 365 (27.9%) | 153 (27.1%) | 0.98 |
|  | Unstable angina | 268 (20.5%) | 115 (20.4%) |  |
|  | Non-STEMI | 369 (28.2%) | 167 (29.6%) |  |
|  | STEMI | 254 (19.4%) | 107 (19.0%) |  |
|  | Other | 52 (4.0%) | 22 (3.9%) |  |
| Contrast volume (ml), mean (SD) |  | 168.7 (77.4) | 150.2 (72.8) | <0.001 |
| Arterial access | Radial | 1234 (94.3%) | 522 (92.6%) | 0.30 |
|  | Femoral | 71 (5.4%) | 41 (7.3%) |  |
|  | Other | 3 (0.2%) | 1 (0.2%) |  |
| Use of intracoronary imaging |  | 239 (18.3%) | 107 (19.0%) | 0.72 |
| Angiographic findings | 1 vessel disease | 859 (65.7%) | 376 (66.7%) | 0.82 |
|  | 2 vessel disease | 340 (26.0%) | 139 (24.6%) |  |
|  | 3 vessel disease and/or left main disease | 109 (8.3%) | 49 (8.7%) |  |
| Number of stents, mean (SD) |  | 1.9 (1.1) | 1.8 (1.2) | 0.053 |
| Complete revascularization |  | 1137 (86.9%) | 498 (88.3%) | 0.41 |
| **Medications prior to PCI** |  |  |  |  |
| ASA |  | 422 (32.3%) | 194 (34.4%) | 0.37 |
| P2Y12 |  | 142 (10.9%) | 59 (10.5%) | 0.80 |
| Statin |  | 410 (31.3%) | 197 (34.9%) | 0.13 |
| Beta-blocker |  | 371 (28.4%) | 183 (32.4%) | 0.076 |
| ARB/ACEi |  | 350 (26.8%) | 157 (27.8%) | 0.63 |
| Calcium channel blocker |  | 183 (14.0%) | 70 (12.4%) | 0.36 |
| **Stent-level** |  | **DES** | **Absorb BRS** | **p-value** |
|  |  | **(n=1522)** | **(n=777)** |  |
| **Lesion characteristics** |  |  |  |  |
| Lesion location | Left main lesion | 6 (0.4%) | 3 (0.4%) | 0.99 |
|  | Other proximal lesion | 534 (35.1%) | 275 (35.4%) |  |
|  | Distal lesion | 982 (64.5%) | 499 (64.2%) |  |
| Lesion classification | Type A | 133 (8.7%) | 73 (9.4%) | 0.84 |
|  | Type B1-B2 | 992 (65.2%) | 511 (65.8%) |  |
|  | Type C or B1-B2 with bifurcation | 396 (26.0%) | 192 (24.7%) |  |
|  | Other | 1 (0.1%) | 1 (0.1%) |  |
| **Stent characteristics** |  |  |  |  |
| Stent diameter (mm), mean (SD) |  | 3.1 (0.5) | 3.2 (0.4) | <0.001 |
| Stent length (mm), mean (SD) |  | 20.8 (8.0) | 19.9 (6.1) | 0.008 |
| Use of postdilation |  | 549 (36.1%) | 502 (64.6%) | <0.001 |
| Stent name | Biosensors BioMatrix | 51 (3.4%) | 0 (0.0%) | <0.001 |
|  | Abbott Xience Prime | 14 (0.9%) | 0 (0.0%) |  |
|  | BS Promus Element | 22 (1.4%) | 0 (0.0%) |  |
|  | Medtronic Resolute Integrity | 353 (23.2%) | 0 (0.0%) |  |
|  | Biotronik Orsiro | 100 (6.6%) | 0 (0.0%) |  |
|  | Abbott ABSORB BVS | 0 (0.0%) | 777 (100.0%) |  |
|  | BS Promus Element Plus | 58 (3.8%) | 0 (0.0%) |  |
|  | Abbott Xience Xpedition | 153 (10.1%) | 0 (0.0%) |  |
|  | BS Promus Premier | 378 (24.8%) | 0 (0.0%) |  |
|  | BS Synergy | 178 (11.7%) | 0 (0.0%) |  |
|  | Terumo Ultimaster | 36 (2.4%) | 0 (0.0%) |  |
|  | Biosensors Biofreedom | 3 (0.2%) | 0 (0.0%) |  |
|  | Medtronic Resolute Onyx | 168 (11.0%) | 0 (0.0%) |  |
|  | Abbott Xience ProX | 8 (0.5%) | 0 (0.0%) |  |
|  |  |  |  |  |

Footnote: Categorical variables: Count(Percentage).

Abbott Xience family: Abbott group, Biosensors family: Biosensors Interventional Technologies Pte Ltd, Biotronic Orsiro: Biotronic group, BS family: Boston scientific (BS) corporation, Medtronic Resolute family: Medtronic PLC, Terumo Ultimaster: Terumo corporation.

**Supplementary Table S2 E**. Baseline characteristics of patient eligible for “Myocardial infarction” landmark analysis

| **Patient-level** |  | **DES** | **Absorb BRS** | **p-value** |
| --- | --- | --- | --- | --- |
|  |  | **(n=1216)** | **(n=509)** |  |
| **Patient demographics** |  |  |  |  |
| Inclusion time | 2011-2013 | 360 (29.6%) | 135 (26.5%) | 0.23 |
|  | 2014 | 462 (38.0%) | 189 (37.1%) |  |
|  | 2015-2018 | 394 (32.4%) | 185 (36.3%) |  |
| Age, mean (SD) |  | 59.7 (10.9) | 59.7 (10.8) | 0.96 |
| Age ≥ 80 |  | 42 (3.5%) | 14 (2.8%) | 0.45 |
| Sex | Men | 998 (82.1%) | 408 (80.2%) | 0.35 |
| Smoking status | Non-smoker | 505 (42.4%) | 214 (43.3%) | 0.26 |
|  | Previous smoker | 400 (33.6%) | 179 (36.2%) |  |
|  | Active smoker | 286 (24.0%) | 101 (20.4%) |  |
| **Comorbidities** |  |  |  |  |
| Diabetes Mellitus |  | 176 (14.5%) | 80 (15.7%) | 0.51 |
| Hypertension |  | 228 (18.8%) | 105 (20.6%) | 0.37 |
| Hyperlipidaemia |  | 493 (40.5%) | 222 (43.6%) | 0.24 |
| Heart failure |  | 21 (1.7%) | 7 (1.4%) | 0.60 |
| Renal failure |  | 4 (0.3%) | 4 (0.8%) | 0.20 |
| Estimated GFR, (ml/min/1,73 m²), mean (SD) |  | 89.0 (20.3) | 89.2 (22.2) | 0.86 |
| Previous stroke |  | 29 (2.4%) | 16 (3.1%) | 0.37 |
| Previous MI |  | 128 (10.5%) | 64 (12.6%) | 0.22 |
| Previous PCI |  | 153 (12.6%) | 76 (14.9%) | 0.19 |
| Previous CABG |  | 20 (1.6%) | 8 (1.6%) | 0.91 |
| **Procedural characteristics** |  |  |  |  |
| Indication | Stable CAD | 355 (29.2%) | 145 (28.5%) | 0.99 |
|  | Unstable angina | 246 (20.2%) | 106 (20.8%) |  |
|  | Non-STEMI | 338 (27.8%) | 146 (28.7%) |  |
|  | STEMI | 230 (18.9%) | 92 (18.1%) |  |
|  | Other | 47 (3.9%) | 20 (3.9%) |  |
| Contrast volume (ml), mean (SD) |  | 167.9 (77.8) | 150.2 (71.2) | <0.001 |
| Arterial access | Radial | 1145 (94.2%) | 473 (92.9%) | 0.58 |
|  | Femoral | 68 (5.6%) | 35 (6.9%) |  |
|  | Other | 3 (0.2%) | 1 (0.2%) |  |
| Use of intracoronary imaging |  | 217 (17.8%) | 100 (19.6%) | 0.38 |
| Angiographic findings | 1 vessel disease | 807 (66.4%) | 342 (67.2%) | 0.79 |
|  | 2 vessel disease | 311 (25.6%) | 123 (24.2%) |  |
|  | 3 vessel disease and/or left main disease | 98 (8.1%) | 44 (8.6%) |  |
| Number of stents, mean (SD) |  | 1.8 (1.1) | 1.8 (1.2) | 0.22 |
| Complete revascularization |  | 1068 (87.8%) | 456 (89.6%) | 0.30 |
| **Medications prior to PCI** |  |  |  |  |
| ASA |  | 398 (32.7%) | 177 (34.8%) | 0.41 |
| P2Y12 |  | 131 (10.8%) | 52 (10.2%) | 0.73 |
| Statin |  | 386 (31.7%) | 179 (35.2%) | 0.17 |
| Beta-blocker |  | 346 (28.5%) | 167 (32.8%) | 0.071 |
| ARB/ACEi |  | 325 (26.7%) | 136 (26.7%) | 1.00 |
| Calcium channel blocker |  | 169 (13.9%) | 65 (12.8%) | 0.53 |
| **Stent-level** |  | **DES** | **Absorb BRS** | **p-value** |
|  |  | **(n=1412)** | **(n=702)** |  |
| **Lesion characteristics** |  |  |  |  |
| Lesion location | Left main lesion | 6 (0.4%) | 3 (0.4%) | 0.88 |
|  | Other proximal lesion | 497 (35.2%) | 255 (36.3%) |  |
|  | Distal lesion | 909 (64.4%) | 444 (63.2%) |  |
| Lesion classification | Type A | 123 (8.7%) | 68 (9.7%) | 0.78 |
|  | Type B1-B2 | 915 (64.8%) | 457 (65.1%) |  |
|  | Type C or B1-B2 with bifurcation | 373 (26.4%) | 176 (25.1%) |  |
|  | Other | 1 (0.1%) | 1 (0.1%) |  |
| **Stent characteristics** |  |  |  |  |
| Stent diameter (mm), mean (SD) |  | 3.1 (0.5) | 3.2 (0.4) | <0.001 |
| Stent length (mm), mean (SD) |  | 20.7 (7.9) | 19.8 (6.1) | 0.014 |
| Use of postdilation |  | 511 (36.2%) | 448 (63.8%) | <0.001 |
| Stent name | Biosensors BioMatrix | 48 (3.4%) | 0 (0.0%) | <0.001 |
|  | Abbott Xience Prime | 14 (1.0%) | 0 (0.0%) |  |
|  | BS Promus Element | 22 (1.6%) | 0 (0.0%) |  |
|  | Medtronic Resolute Integrity | 319 (22.6%) | 0 (0.0%) |  |
|  | Biotronik Orsiro | 92 (6.5%) | 0 (0.0%) |  |
|  | Abbott ABSORB BVS | 0 (0.0%) | 702 (100.0%) |  |
|  | BS Promus Element Plus | 55 (3.9%) | 0 (0.0%) |  |
|  | Abbott Xience Xpedition | 144 (10.2%) | 0 (0.0%) |  |
|  | BS Promus Premier | 358 (25.4%) | 0 (0.0%) |  |
|  | BS Synergy | 165 (11.7%) | 0 (0.0%) |  |
|  | Terumo Ultimaster | 34 (2.4%) | 0 (0.0%) |  |
|  | Biosensors Biofreedom | 2 (0.1%) | 0 (0.0%) |  |
|  | Medtronic Resolute Onyx | 153 (10.8%) | 0 (0.0%) |  |
|  | Abbott Xience ProX | 6 (0.4%) | 0 (0.0%) |  |
|  |  |  |  |  |

Footnote: Categorical variables: Count(Percentage).

Abbott Xience family: Abbott group, Biosensors family: Biosensors Interventional Technologies Pte Ltd, Biotronic Orsiro: Biotronic group, BS family: Boston scientific (BS) corporation, Medtronic Resolute family: Medtronic PLC, Terumo Ultimaster: Terumo corporation.

**Supplementary Table S2 F**. Baseline characteristics of patient eligible for “Any restenosis” landmark analysis

| **Patient-level** |  | **DES** | **Absorb BRS** | **p-value** |
| --- | --- | --- | --- | --- |
|  |  | **(n=1273)** | **(n=540)** |  |
| **Patient demographics** |  |  |  |  |
| Inclusion time | 2011-2013 | 371 (29.1%) | 145 (26.9%) | 0.33 |
|  | 2014 | 491 (38.6%) | 202 (37.4%) |  |
|  | 2015-2018 | 411 (32.3%) | 193 (35.7%) |  |
| Age, mean (SD) |  | 59.7 (11.0) | 59.8 (10.9) | 0.89 |
| Age ≥ 80 |  | 43 (3.4%) | 16 (3.0%) | 0.65 |
| Sex | Men | 1042 (81.9%) | 436 (80.7%) | 0.58 |
| Smoking status | Non-smoker | 518 (41.7%) | 225 (42.9%) | 0.23 |
|  | Previous smoker | 417 (33.6%) | 189 (36.1%) |  |
|  | Active smoker | 307 (24.7%) | 110 (21.0%) |  |
| **Comorbidities** |  |  |  |  |
| Diabetes Mellitus |  | 194 (15.2%) | 86 (15.9%) | 0.71 |
| Hypertension |  | 248 (19.5%) | 117 (21.7%) | 0.29 |
| Hyperlipidaemia |  | 515 (40.5%) | 234 (43.3%) | 0.26 |
| Heart failure |  | 21 (1.6%) | 12 (2.2%) | 0.40 |
| Renal failure |  | 6 (0.5%) | 5 (0.9%) | 0.25 |
| Estimated GFR, (ml/min/1,73 m²), mean (SD) |  | 88.9 (20.7) | 88.8 (22.0) | 0.94 |
| Previous stroke |  | 33 (2.6%) | 17 (3.1%) | 0.51 |
| Previous MI |  | 135 (10.6%) | 71 (13.1%) | 0.12 |
| Previous PCI |  | 159 (12.5%) | 79 (14.6%) | 0.22 |
| Previous CABG |  | 21 (1.6%) | 8 (1.5%) | 0.79 |
| **Procedural characteristics** |  |  |  |  |
| Indication | Stable CAD | 356 (28.0%) | 146 (27.0%) | 0.98 |
|  | Unstable angina | 256 (20.1%) | 111 (20.6%) |  |
|  | Non-STEMI | 357 (28.0%) | 158 (29.3%) |  |
|  | STEMI | 252 (19.8%) | 104 (19.3%) |  |
|  | Other | 52 (4.1%) | 21 (3.9%) |  |
| Contrast volume (ml), mean (SD) |  | 168.4 (77.0) | 148.2 (68.9) | <0.001 |
| Arterial access | Radial | 1201 (94.3%) | 501 (92.8%) | 0.40 |
|  | Femoral | 69 (5.4%) | 38 (7.0%) |  |
|  | Other | 3 (0.2%) | 1 (0.2%) |  |
| Use of intracoronary imaging |  | 234 (18.4%) | 103 (19.1%) | 0.73 |
| Angiographic findings | 1 vessel disease | 842 (66.1%) | 365 (67.6%) | 0.67 |
|  | 2 vessel disease | 329 (25.8%) | 129 (23.9%) |  |
|  | 3 vessel disease and/or left main disease | 102 (8.0%) | 46 (8.5%) |  |
| Number of stents, mean (SD) |  | 1.8 (1.1) | 1.7 (1.1) | 0.022 |
| Complete revascularization |  | 1110 (87.2%) | 481 (89.1%) | 0.26 |
| **Medications prior to PCI** |  |  |  |  |
| ASA |  | 408 (32.1%) | 184 (34.1%) | 0.40 |
| P2Y12 |  | 137 (10.8%) | 56 (10.4%) | 0.80 |
| Statin |  | 392 (30.8%) | 187 (34.6%) | 0.11 |
| Beta-blocker |  | 358 (28.1%) | 173 (32.0%) | 0.094 |
| ARB/ACEi |  | 334 (26.2%) | 148 (27.4%) | 0.61 |
| Calcium channel blocker |  | 177 (13.9%) | 67 (12.4%) | 0.39 |
| **Stent-level** |  | **DES** | **Absorb BRS** | **p-value** |
|  |  | **(n=1475)** | **(n=736)** |  |
| **Lesion characteristics** |  |  |  |  |
| Lesion location | Left main lesion | 6 (0.4%) | 2 (0.3%) | 0.88 |
|  | Other proximal lesion | 517 (35.1%) | 258 (35.1%) |  |
|  | Distal lesion | 952 (64.5%) | 476 (64.7%) |  |
| Lesion classification | Type A | 130 (8.8%) | 70 (9.5%) | 0.86 |
|  | Type B1-B2 | 965 (65.4%) | 484 (65.8%) |  |
|  | Type C or B1-B2 with bifurcation | 379 (25.7%) | 181 (24.6%) |  |
|  | Other | 1 (0.1%) | 1 (0.1%) |  |
| **Stent characteristics** |  |  |  |  |
| Stent diameter (mm), mean (SD) |  | 3.1 (0.5) | 3.3 (0.4) | <0.001 |
| Stent length (mm), mean (SD) |  | 20.8 (8.0) | 19.8 (6.0) | 0.004 |
| Use of postdilation |  | 524 (35.5%) | 468 (63.6%) | <0.001 |
| Stent name | Biosensors BioMatrix | 49 (3.3%) | 0 (0.0%) | <0.001 |
|  | Abbott Xience Prime | 14 (0.9%) | 0 (0.0%) |  |
|  | BS Promus Element | 21 (1.4%) | 0 (0.0%) |  |
|  | Medtronic Resolute Integrity | 341 (23.1%) | 0 (0.0%) |  |
|  | Biotronik Orsiro | 98 (6.6%) | 0 (0.0%) |  |
|  | Abbott ABSORB BVS | 0 (0.0%) | 736 (100.0%) |  |
|  | BS Promus Element Plus | 57 (3.9%) | 0 (0.0%) |  |
|  | Abbott Xience Xpedition | 150 (10.2%) | 0 (0.0%) |  |
|  | BS Promus Premier | 367 (24.9%) | 0 (0.0%) |  |
|  | BS Synergy | 175 (11.9%) | 0 (0.0%) |  |
|  | Terumo Ultimaster | 33 (2.2%) | 0 (0.0%) |  |
|  | Biosensors Biofreedom | 3 (0.2%) | 0 (0.0%) |  |
|  | Medtronic Resolute Onyx | 159 (10.8%) | 0 (0.0%) |  |
|  | Abbott Xience ProX | 8 (0.5%) | 0 (0.0%) |  |
|  |  |  |  |  |

Footnote: Categorical variables: Count(Percentage).

Abbott Xience family: Abbott group, Biosensors family: Biosensors Interventional Technologies Pte Ltd, Biotronic Orsiro: Biotronic group, BS family: Boston scientific (BS) corporation, Medtronic Resolute family: Medtronic PLC, Terumo Ultimaster: Terumo corporation.
